# Nuclear Factor κB-COX2 Pathway Activation in Non-myelinating Schwann Cells Is Necessary for the Maintenance of Neuropathic Pain *in vivo*

**DOI:** 10.3389/fncel.2021.782275

**Published:** 2022-01-14

**Authors:** Alison Xiaoqiao Xie, Sarah Taves, Ken McCarthy

**Affiliations:** ^1^Department of Surgery, University of Colorado Anschutz Medical Campus, Aurora, CO, United States; ^2^Department of Pharmacology, University of North Carolina at Chapel Hill, Chapel Hill, NC, United States

**Keywords:** GFAP-positive glia, peripheral nervous system, non-myelinating Schwann cells, nuclear factor κB signaling, COX2, neuropathic pain, tetracycline transactivator system

## Abstract

Chronic neuropathic pain leads to long-term changes in the sensitivity of both peripheral and central nociceptive neurons. Glial fibrillary acidic protein (GFAP)-positive glial cells are closely associated with the nociceptive neurons including astrocytes in the central nervous system (CNS), satellite glial cells (SGCs) in the sensory ganglia, and non-myelinating Schwann cells (NMSCs) in the peripheral nerves. Central and peripheral GFAP-positive cells are involved in the maintenance of chronic pain through a host of inflammatory cytokines, many of which are under control of the transcription factor nuclear factor κB (NFκB) and the enzyme cyclooxygenase 2 (COX2). To test the hypothesis that inhibiting GFAP-positive glial signaling alleviates chronic pain, we used (1) a conditional knockout (cKO) mouse expressing Cre recombinase under the hGFAP promoter and a floxed COX2 gene to inactivate the COX2 gene specifically in GFAP-positive cells; and (2) a tet-Off tetracycline transactivator system to suppress NFκB activation in GFAP-positive cells. We found that neuropathic pain behavior following spared nerve injury (SNI) significantly decreased in COX2 cKO mice as well as in mice with decreased glial NFκB signaling. Additionally, experiments were performed to determine whether central or peripheral glial NFκB signaling contributes to the maintenance of chronic pain behavior following nerve injury. Oxytetracycline (Oxy), a blood-brain barrier impermeable analog of doxycycline was employed to restrict transgene expression to CNS glia only, leaving peripheral glial signaling intact. Signaling inactivation in central GFAP-positive glia alone failed to exhibit the same analgesic effects as previously observed in animals with both central and peripheral glial signaling inhibition. These data suggest that the NFκB-COX2 signaling pathway in NMSCs is necessary for the maintenance of neuropathic pain *in vivo*.

## Introduction

Neuropathic pain resulting from peripheral nerve insult is the result of both peripheral and central sensitization of nociceptive neurons. Both central and peripheral GFAP-positive glia have also been implied in neuropathic pain through the release of inflammatory molecules (Ma and Bisby, 1998; Milligan and Watkins, 2009; Fu et al., 2010). Activated glia produce proinflammatory cytokines, such as interleukin-1β (IL-1β) and tumor necrosis factor α (TNFα), which exert at least part of their effect through the activation of the transcription factor nuclear factor kappa B (NFκB; Neill and Kaltschmidt, 1997; Niederberger et al., 2007). It has been shown that inhibition of NFκB in GFAP-positive glia effectively reduced hyperalgesia in animal models of chronic pain, including the formalin model of inflammatory pain and the chronic constriction injury model of neuropathic pain (Fu et al., 2007, 2010; Meunier et al., 2007).

NFκB positively regulates the expression of several proinflammatory cytokines, including nitric oxide and dynorphin as well as increasing the expression of the enzyme cyclooxygenase-2 (COX2; Adcock et al., 1997; Neill and Kaltschmidt, 1997). The major downstream product of COX2 activation is prostaglandin E2 (PGE2). Peripheral or intrathecal injection of PGE2 causes pronounced hyperalgesia (Ferreira, 1972; Ferreira et al., 1978). However, systemic administration of COX2 inhibitors performs better than intrathecal COX2 inhibitors at decreasing pain behavior (Takeda et al., 2005). While COX2 seems to be involved in the generation of persistent pain, the cellular source of the COX2 upregulation remains unidentified.

In this study, we aimed to use transgenic mouse models to dissect the cellular origins of glial-produced inflammatory mediators, such as COX2. Two mouse lines were chosen to manipulate the NFκB-COX2 inflammatory pathways in GFAP-positive glia. First, a conditional transgenic mouse line expressing Cre recombinase under the hGFAP promoter was bred to floxed-COX2 mice to inactivate the COX2 gene specifically in GFAP-positive cells. Second, a mouse line expressing dominant negative version of IKKβ driven by the tetO promoter (IKKdn mice) (Huber et al., 2002) was crossed to a line carrying the tetracycline transactivator (tTA) driven by the hGFAP promoter, resulting in selective inactivation of NFκB signaling in GFAP-positive glial cells. The hGFAP promoter drives gene expression in both astrocytes in the CNS and non-myelinating Schwann cells (NMSCs). Therefore, experiments were conducted to tease apart the contribution of the peripheral versus central GFAP-positive glial signaling in neuropathic pain in this study as well.

## Materials and Methods

### Mice

All procedures and behavioral experiments involving animals were approved by the Institutional Animal Care and Use Committee at the University of North Carolina at Chapel Hill. Mice were raised under a 12:12 light:dark cycle and fed Prolab RMH (LabDiet) *ad libitum*. All mice were backcrossed to C57BL6/J for greater than ten generations. Young adult mice between postnatal day (P)60 to P100 were used in all experiments.

### Generation of Glial Fibrillary Acidic Protein-Specific COX2-Deficient Mice

Floxed-COX2 mice were generated as previously described (Yu et al., 2006). Briefly, floxed-COX2 mice were generated by inserting *loxP* sites, using homologous recombination, into introns 5 and 8 to enable deletion of exons 6–8. The insertion of the two *loxP* sites did not affect normal COX2 expression and induction (Yu et al., 2006; Vardeh et al., 2009). GFAP-CreERT2 mice were generated previously in our lab (Casper et al., 2007). Briefly, the human 2.2 kB GFAP promoter from pGfa2lac1 (M. Brenner, UAB) was cloned upstream of intron from β-globin. CreERT2 was excised from pCreERT2 (P. Chambon, Collège de France) and cloned behind β-globin and upstream of SV40 pA. Heterozygous GFAP-CreERT mice were bred to homozygous floxed-COX2 mice to produce GFAP-CreERT::floxed-COX2 mice (experimental group) and Cre-negative, floxed-COX2 mice (littermate controls). The GFAP-CreERT::floxed COX2 mice were induced with a course of 16 tamoxifen injections (3 mg/40 g), twice daily I.P., starting at post-natal day (p) 21. Littermate mice were given tamoxifen to control for the potential effect of tamoxifen injections as well.

### Generation of Glial Fibrillary Acidic Protein-Specific NFκB Suppressor Mice

Another transgenic mouse line, the tetO-IKKdn mice were generated as previously described (Herrmann et al., 2005). Briefly, a transdominant version of human IKK2 (VSV-IKK2-DN; D145N) was cloned into the bidirectional tetracycline-dependent expression vector pBl-5. tetO-IKKdn mice were crossed with mice in which the tTA is driven by a 2.2 kb fragment of the human GFAP promoter (Casper et al., 2007). The GFAP-tTA::tetO-IKK2dn mice (IKKdn mice) were maintained on doxycycline (25 μg/mL in the drinking water) to suppress transgene expression throughout the central and peripheral nervous system while *in utero* and until p21. At weaning, the mice were taken off doxycycline and given drinking water either without doxycycline (to allow IKKdn expression both central and peripheral GFAP-positive cells) or with Oxy (200 mg/mL) to suppress IKKdn expression only in peripheral GFAP-positive glia. The mice were maintained on this treatment for at least 30 days and until experimental use.

### Spared Nerve Injury Model of Neuropathic Pain

Neuropathic pain was generated using a model of spared nerve injury (SNI) adapted from Dr. Allan Basbaum’s laboratory (Shields et al., 2003). Using isoflurane, mice at least 60 days old were deeply anesthetized, as monitored by toe pinch and eye blink reflexes. The left hind leg was secured with tape, the hair removed with Nair, and the leg was cleaned with iodine and alcohol. The three divisions of the sciatic nerve were exposed. The common peroneal and sural nerves were ligated, and a 1–3 mm portion of the nerve was removed distal to the ligation to prevent regrowth. The musculature was returned to its original position, and the skin closed with wound clips. The area was again cleaned with iodine and alcohol, and anti-bacterial and anti-fungal agents were applied. The animals were housed in cages with soft bedding.

### Chronic Pain Behavior Assessment Using Von Frey Tests

Animals were singly placed in a Plexiglas enclosure on top of a metal grate. Paw withdrawal frequency was used to measure mechanical allodynia in animal hind paws. A Von Frey hair (IITC Life Science, Woodland Hills, CA, United States) calibrated to 3.61 log force was applied to the center of the foot pad (the region innervated by the tibial nerve) ten times at an interval of at least 1 min between applications. Both hind paws were tested; the mechanical sensitivity of the contralateral paw to the surgery side was used as an internal control for the sensitivity of the ipsilateral paw. The number of times the paw was withdrawn due to the stimulus was recorded. The paw withdrawal threshold was measured using the up-down method of von Frey hair application (Chaplan et al., 1994). Mechanical sensitivity was measured for 2 days prior to surgery as the baseline of hind paw mechanical sensitivity. Von Frey tests were repeated post-SNI at 4 days, 7 days, and then every subsequent week through 3 weeks.

### Immunohistochemistry

Animals were deeply anesthetized with an intraperitoneal injection of urethane (2 mg/kg). Minimally five animals were used in each experimental group as biological replicates. Once deeply anesthetized, as monitored by the toe-pinch and eye-blink reflexes, the animals were transcardially perfused with 4% paraformaldehyde (PFA) in phosphate buffered saline (PBS). Lumbar segments 4–6 (L4–L6) of the spinal cord, the corresponding dorsal root ganglia (DRG) and a 5–8 mm segment of the tibial nerve (at the level of the lesion in the other branches) were removed and post-fixed in ice-cold 4% PFA for 2 h. The tissue was cryoprotected with 30% sucrose in PBS and subsequently frozen in OCT. Fourteen micrometer (14 μm) thick sections were made on a cryostat (Leica, Wetzlar, Germany). Immunohistochemistry was performed with primary antibodies against mouse GFAP (1:500; Dako), chicken GFAP (1:500; Abcam), rabbit Iba-1 (1:500; Wako), mouse NeuN (1:500; Millipore), rabbit peripherin (1:1000; Millipore), rabbit neurofilament 200 (1:500; Sigma), mouse S100β (1:1000; Sigma), rabbit pNFκB (1:400; Cell Signaling) and biotinylated IB4 lectin (1:100; Vector). Secondary antibodies were either anti-mouse, anti-rabbit, or anti-chicken Alexa 568, Alexa 488, Alexa 647, or avidin-conjugated Alexa 568. The slides were cover-slipped with Vectashield containing DAPI. Images were collected on a Zeiss Axioscope fluorescent microscope with a Dage XL16 camera or on a Zeiss LSM 710 confocal microscope. Quantification of immunopositivity was performed manually.

### Statistical Analysis

All data are presented as the mean ± SEM. Significant differences were assessed by Student’s *t*-test or, in the case of behavior assays, a two-way repeated measures analysis of variance followed by a Bonferroni *post hoc* analysis. Indicators of significance were as follows: *P* < 0.05 (*), *P* < 0.01 (^**^), and *P* < 0.001 (^***^).

## Results

### Glial-Specific Transgenic COX2 Knockout Reduces Mechanical Sensitivity Following Peripheral Nerve Injury

Prostaglandin E2 has been shown to sensitize nociceptive neurons (Ferreira, 1972; Ferreira et al., 1978). In this experiment, we used tamoxifen inducible GFAP-CreERT2 mice to drive the removal of the floxed COX2 gene specifically in GFAP-positive glia. Tamoxifen injections were given post-weaning to avoid potential developmental confounds of transgene expression. To control for possible effects of tamoxifen, a subset of Cre-negative littermate controls was also injected with tamoxifen along with the experimental group.

**FIGURE 1:**
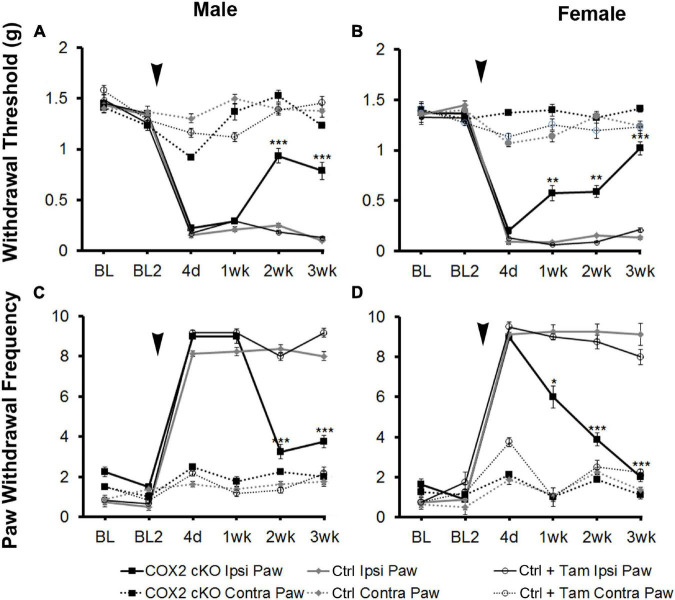
SNI induced long-term mechanical allodynia that was temporarily alleviated in both male and female COX2 cKO mice. Withdrawal threshold **(A,B)** and paw withdrawal frequency **(C,D)** following SNI in male **(A,C)** and female **(B,D)** COX2 cKO mice (male *n* = 10; female *n* = 10), littermate controls (male *n* = 10; female *n* = 10), and littermate animals given tamoxifen (male *n* = 10; female *n* = 10) were shown. In COX2 cKO mice and a subset of littermate controls, 16 tamoxifen injections (3 mg/40 g) were given over a 8-day period (twice daily I.P., starting at p21). SNI were performed at P60. There was no difference in basal mechanical sensitivity between any of the groups. Bonferroni *post hoc* analysis: *P < 0.05, **P < 0.01, ***P < 0.001, for COX2 cKO vs. control ipsilateral hind paws. Black arrows indicate the time of spared nerve injury.

To assess the role of COX2 in GFAP-positive glial cells on pain behavior, we measured mechanical allodynia and paw withdrawal threshold for 2 days prior to SNI and for 3 weeks following peripheral nerve injury in COX2 cKO, littermate controls, and tamoxifen (Tam)-injected littermate controls (male COX2 cKO *n* = 10, female COX2 cKO *n* = 10, male controls *n* = 10, female controls *n* = 10, male control + Tam *n* = 10, female control + Tam *n* = 10). As shown in Figure 1, prior to SNI, no significant differences in mechanical sensitivity at the baseline were detected among different genotypes. At 4 days post-SNI, both male and female animals developed mechanical allodynia in the paw ipsilateral to the lesion, but not in the contralateral paw, as indicated by increased paw withdrawal frequency and decreased withdrawal threshold. In littermate control and tamoxifen injected littermate control animals, mechanical sensitivity of the ipsilateral paw was maintained through the 3-week testing period. By 2 weeks post-injury, both male and female COX2 cKO mice showed abrupt and profound reductions in mechanical sensitivity (*P* < 0.001 compared to the ipsilateral paw of the littermate control animals). No significant impact of tamoxifen injections alone was observed on the littermate control animals at any time point. These data suggested that COX2 in GFAP-positive glia contributes to the maintenance of neuropathic pain following peripheral nerve injury.

### Glial-Specific Transgenic Suppression of NFκB Reduces Mechanical Sensitivity Following Peripheral Nerve Injury

**FIGURE 2:**
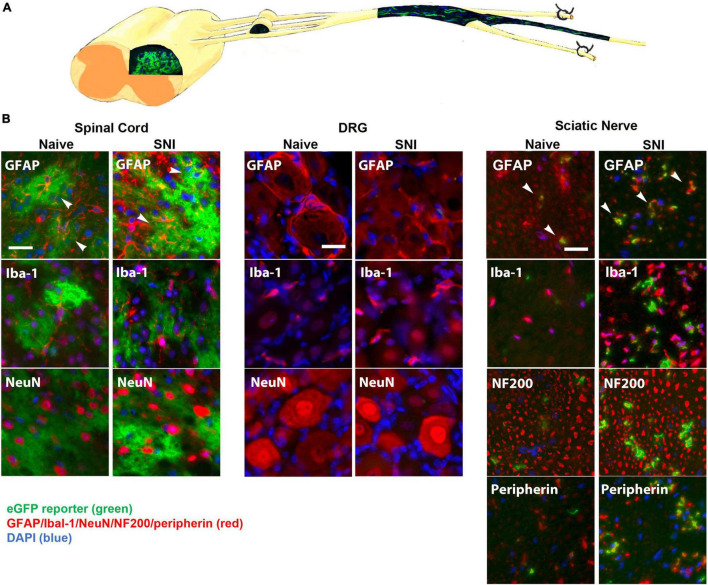
**(A)** A diagram showing expression of eGFP in the spinal cord and sciatic nerve (longitudinal section) from GFAP-tTA eGFP reporter mice. The mice were taken off doxycycline at weaning to allow the expression of the transgene in GFAP-positive cells. **(B)** The native eGFP reporter (*green*) colocalized with immunohistochemical staining for astrocytes (GFAP, *red*) in the dorsal horn and with NMSCs (GFAP, *red*) in the sciatic nerve (transverse sections), but not with satellite cells (GFAP, *red*) in the DRG (*N* = 5). eGFP did not colocalize with any markers for microglia or macrophages (Iba-1), neurons (NeuN) or large or small axonal markers (NF200 or peripherin). All cellular and axonal markers are red and are listed on the individual images. Native eGFP is shown in green. DAPI is shown in blue. White arrows indicate colocalized cells. The scale bar is 20 μm.

We next tested the hypothesis that inhibiting NFκB signaling may suppress neuropathic pain *in vivo*. In its inactive form, NFκB is sequestered in the cytoplasm by binding to inhibitory factors of the IκB family. Upon stimulation by inflammatory mediators, IκB is phosphorylated by the IκB kinase (IKKβ) complex, leading to its ubiquitination and degradation, which allows NFκB to translocate to the nucleus. In the nucleus, NFκB promotes the transcription of cytokines, chemokines and proinflammatory molecules. To suppress NFκB signaling, we used the tet-Off system driven by the hGFAP promoter to induce glial-specific expression of a dominant negative form of IKK. To characterize the potential expression in the GFAP-positive glia, GFAP-tTA mice were first bred to a tetO-eGFP reporter line of mice. We observed strong eGFP expression in the dorsal horn, which colocalized with the astrocyte marker GFAP, but not with markers for microglia (Iba-1) or neurons (NeuN) (Figure 2). In the sciatic nerve, we observed strong eGFP expression in GFAP-positive NMSCs. eGFP did not colocalize with markers for macrophages (Iba-1), large diameter axons (NF200) or small diameter axons (peripherin) (Figure 2). Surprisingly, there was no eGFP expression in GFAP-positive SGCs in the dorsal root ganglia (DRG) nor in any other cells of the DRG. Immunohistochemistry was also performed 2 weeks post-SNI to detect potential changes in eGFP localization after nerve lesion and gliosis; however, the expression pattern of eGFP reveal the same pattern in GFAP-positive spinal astrocytes and NMSCs, but not in SGCs (Figure 2).

**FIGURE 3:**
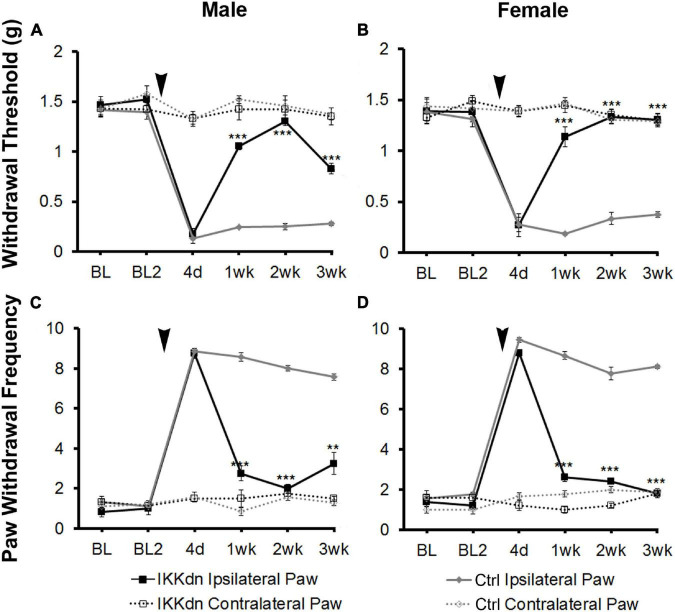
SNI induced long-term mechanical allodynia which was temporarily alleviated in both male and female IKKdn mice. The mice were off doxycycline starting at weaning to allow the expression of the IKKdn in GFAP-positive cells. Withdrawal threshold **(A,B)** and paw withdrawal frequency **(C,D)** following SNI in male **(A,C)** and female **(B,D)** IKKdn mice (male *n* = 6; female *n* = 5) and IKKdn-negative littermate controls (male *n* = 9; female *n* = 9). There was no difference in basal mechanical sensitivity between the groups. Bonferroni *post hoc* analysis: **P < 0.01, ***P < 0.001, IKKdn vs. control ipsilateral hind paws. Black arrows indicate the time of spared nerve injury.

To investigate the necessity of NFκB signaling in GFAP-positive glia on pain behavior, we measured mechanical allodynia and paw withdrawal threshold for 2 days prior to SNI, and for 3 weeks following peripheral nerve injury in IKKdn and IKKdn-negative littermate control animals (male IKKdn *n* = 6, female IKKdn *n* = 5, male littermate control *n* = 9, female littermate control *n* = 9). There was no difference in basal mechanical sensitivity in the male or female groups (Figure 3). Following SNI, all animals developed mechanical allodynia ipsilateral to the nerve lesion, as shown by increased paw withdrawal frequency and decreased withdrawal threshold. In control animals, mechanical sensitivity of the ipsilateral paw was maintained through the 3-week testing period. In IKKdn-positive animals, pain behavior began to subside 1 week post-injury (*P* < 0.001) and was alleviated by 2 weeks post-injury in both the male and female IKKdn mice (*P* < 0.001). No change was observed in the contralateral paws of the IKKdn mice or littermate controls.

### Oxytetracycline (Oxy) Suppresses Peripheral Transgene Expression Using the Tet-Off System While Leaving Central Expression Intact

Persistent pain is associated with ongoing neural plasticity in both primary sensory neurons and spinal dorsal horn neurons. Neurons and glial cells in both peripheral and central sensory nervous system contribute to sensitization underlying persistent pain. Likewise, using the GFAP promoter, our system drives the expression of transgenes in GFAP-positive glia in both CNS (astrocytes) and PNS (NMSCs). The tet-Off system is typically regulated by the tetracycline derivative doxycycline, which, similar to tetracycline, binds the tTA, preventing it from binding to the tetO promoter and blocking transcription of the downstream transgene. Doxycycline is highly bioavailable, and easily crosses the blood-brain barrier. To tease apart the central and peripheral roles of GFAP-positive glia, we used Oxy, a tetracycline derivative that does not cross the blood-brain barrier (Barza et al., 1975) to block the transcription of transgene in peripheral NMSCs but not in central astrocytes.

**FIGURE 4:**
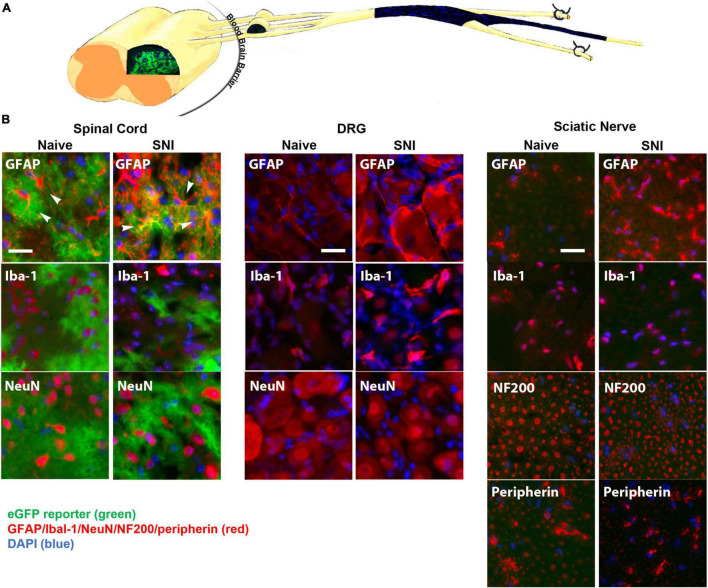
**(A)** A diagram showing expression of eGFP in the spinal cord, but not in the DRG or sciatic nerve (longitudinal section) of GFAP-tTA eGFP reporter mice following treatment with Oxy (starting at weaning). **(B)** Following treatment with Oxy, the native eGFP reporter (*green*) colocalized with immunohistochemical staining for astrocytes (GFAP, *red*) in the dorsal horn, but with not NMSCs (GFAP, *red*) in the sciatic nerve (transverse sections) or with satellite cells (GFAP, *red*) in the DRG (*N* = 5). eGFP did not colocalize with any markers for microglia or macrophages (Iba-1), neurons (NeuN) or large or small axonal markers (NF200 or peripherin). All cellular and axonal markers are shown in red and are listed on the individual images. Native eGFP is shown in green. DAPI is shown in blue. White arrows indicate colocalized cells. The scale bar is 20 μm.

The GFAP-tTA::tetO-eGFP reporter mice were administered Oxy in their drinking water from weaning until p60. At p60, they were sacrificed to examine transgenic protein expression using immunohistochemistry. We found that Oxy effectively inhibited transgenic eGFP expression peripherally while leaving central expression intact (Figure 4) (*n* = 3 per group). There was no eGFP detected in the sciatic nerve either in naïve animals or in animals 2 weeks post-SNI. In the spinal cord, eGFP remained colocalized with the astrocyte marker GFAP in both naïve and nerve-injured animals. eGFP was not colocalized with any markers for microglia or macrophages (Iba-1), neurons (NeuN) or large or small peripheral nerves (NF200 or peripherin).

**FIGURE 5:**
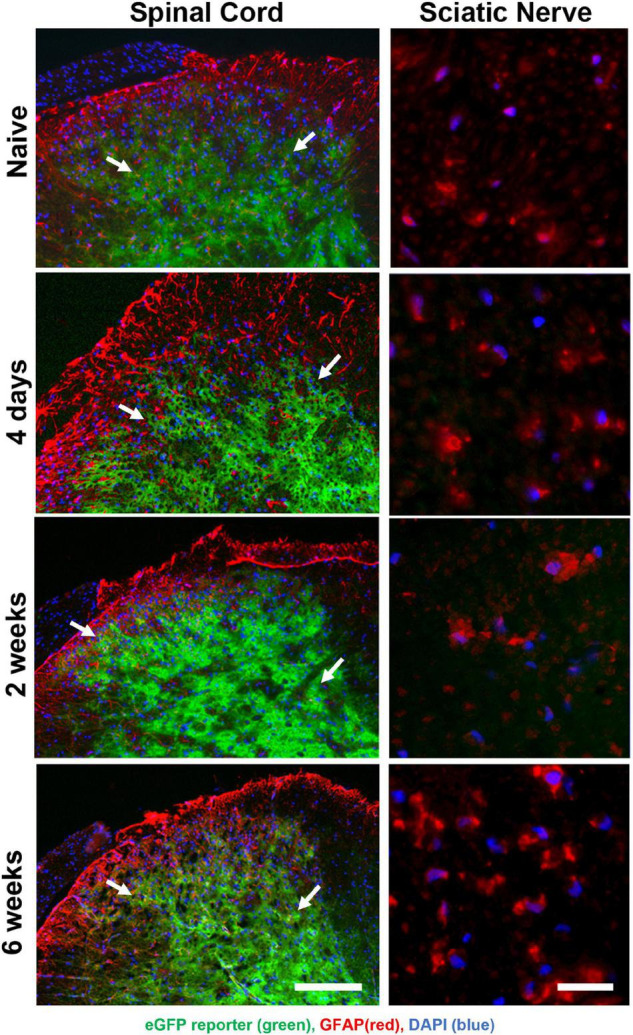
Oxy did not cross the blood-brain barrier to inhibit central reporter gene expression at any time-point following surgery, while it continually suppressed peripheral transgene expression. eGFP expression (*green*) were detected in spinal cord sections (left panels) but not in peripheral sciatic nerve (right panels) and showed cellular colocalization with GFAP immunohistochemical staining (*red*) from mice on Oxy (white arrows) (starting at weaning, *N* = 5). The scale bar on left is 100 μm. The scale bar on right is 20 μm.

The permeability of the blood-brain barrier increases for 1 week post-nerve injury (Beggs et al., 2010). To determine whether Oxy entered the spinal cord in a great enough quantity to regulate transgene expression, we performed time-course of immunohistochemical staining following SNI (Figure 5). Adult animals on Oxy for at least 1 month prior to surgery underwent SNI and were sacrificed at 4 days, 2 weeks, or 6 weeks post-injury (*n* = 3 per time point). eGFP expression was suppressed in all sciatic nerve sections, while its central expression in the spinal cord was preserved at every time point (Figure 5). These findings indicate that Oxy was not able to cross the blood-brain barrier in sufficient concentration to block gene expression post-nerve injury during the experimental period.

Next, we evaluated the ability of Oxy to regulate the expression of IKKdn by examining the downstream marker pNFκB. If effective, Oxy should peripherally inhibit IKKdn expression, thus allowing pNFκB activation in NMSCs of the sciatic nerve. Activated phosphorylated NFκB is localized to the cell nucleus, whereas the non-myelinating Schwann cell marker GFAP is often localized to cellular processes, making colocalization of these two markers difficult. S100β is a marker for both myelinating and non-myelinating Schwann cells and fills the cytosol of the cell, allowing for colocalization with nuclear markers. We, therefore, performed immunohistochemical staining with the markers for GFAP and S100β in conjunction with the nuclear marker DAPI and the pNFκB antibody. Only cells positive for both GFAP and S100β were counted as NMSCs, and only cells with pNFκB localized in the nucleus were counted as pNFκB positive.

**FIGURE 6:**
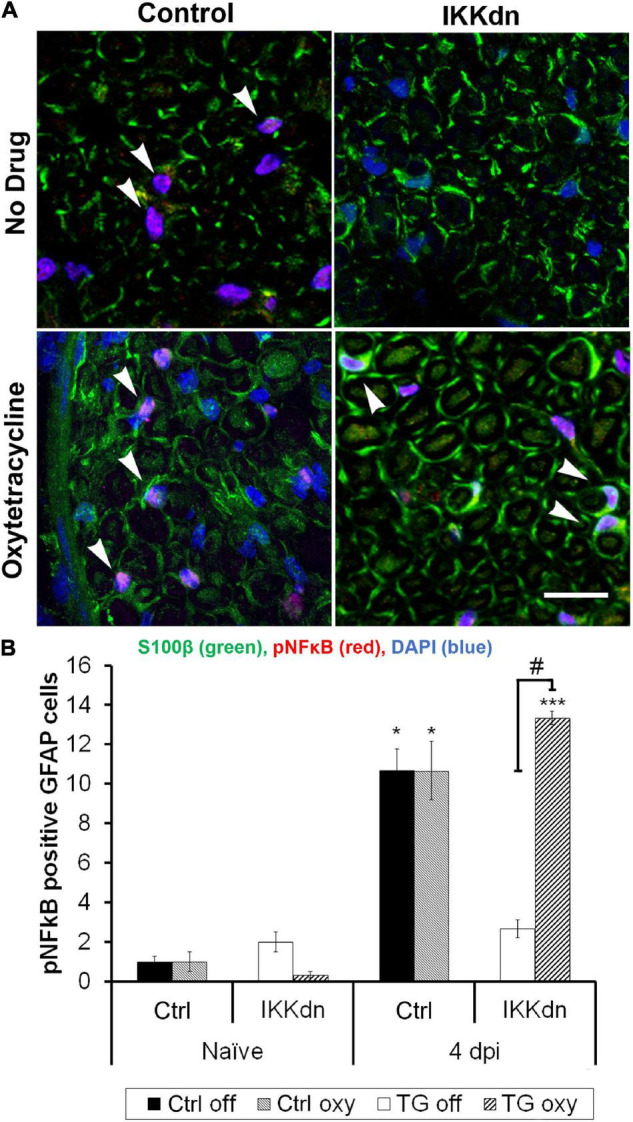
**(A)** Immunostaining for S100β (green), pNFκB (red), and DAPI (blue) in the sciatic nerve 4 days post-SNI. **(B)** Cells positive for GFAP, S100β and nuclear pNFκB were quantified (In all groups, nine images were analyzed from three different animals). The number of positive cells displayed is the mean per image ± SEM. *P < 0.05, ***P < 0.001 for the comparison of the SNI group to its naïve counterpart. #*P* < 0.001 comparing IKKdn 4 days post-injury off drug vs. on Oxy. White arrows indicate colocalized cells.

Quantification was performed in transgenic animals and littermate controls, both on and off Oxy. Prior to nerve injury, animals from all treatment groups displayed little pNFκB staining and very few pNFκB positive NMSCs (cells containing all four colocalized markers) (Figure 6B) (*n* = 3 animals per group, three sections per animal). To drive the expression of pNFκB, we performed SNI and sacrificed the animals 4 days post-injury. Figure 6A shows representative sections from all four treatments at the 4 days post-SNI time point. Following injury, littermate control animals showed increases in nuclear pNFκB staining in NMSCs both in the drug-free and Oxy groups (*P* < 0.05). In transgenic animals, expression of IKKdn completely prevented the SNI-induced increase in pNFκB-positive NMSCs observed in littermate control animals. This transgenic suppression of NFκB activation in the sciatic nerve was eliminated by Oxy administration. IKKdn animals on Oxy showed significantly higher numbers of pNFκB positive NMSCs compared to IKKdn animals without Oxy (*P* < 0.001).

These data showed that Oxy effectively inhibited peripheral transgene expression while leaving CNS glial expression intact. The peripheral expression of IKKdn suppressed NFκB signaling in NMSCs. The administration of Oxy inhibited peripheral expression of IKKdn, allowing NFκB signaling to occur. Therefore, in IKKdn animals on Oxy, IKKdn should only inhibit NFκB signaling centrally in astrocytes.

### Astrocyte-Specific Transgenic Inhibition of NFκB Has No Effect on Mechanical Sensitivity Following Peripheral Nerve Injury

**FIGURE 7:**
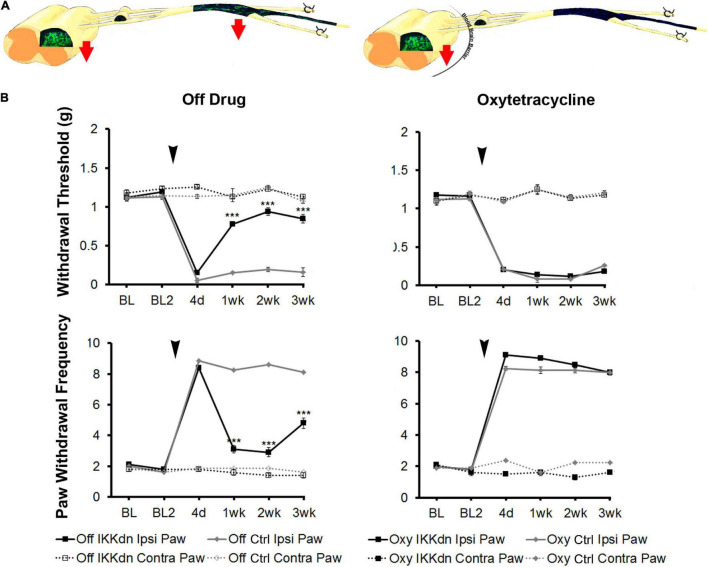
**(A)** Diagrams showing expression of eGFP from GFAP-tTA::eGFP animals with or without Oxy treatment. Mice were taken off doxycycline at weaning followed by drinking water (to allow the expression of the transgene in GFAP-positive cells) or drinking water with Oxy (to only express IKKdn in peripheral GFAP-positive glia). The red arrows indicate the regions of transgene expression and thus the regions of NFκB suppression. **(B)** Peripheral suppression of IKKdn by Oxy eliminated the IKKdn phenotype following SNI. Withdrawal threshold (top panels) and paw withdrawal frequency (bottom panels) following SNI in IKKdn and littermate control animals with and without administration of the transgene inhibitor Oxy (IKKdn Off *n* = 10; IKKdn Oxy *n* = 10; control Off *n* = 8; control Oxy *n* = 9). Bonferroni *post hoc* analysis: ***P < 0.001, for IKKdn vs. control ipsilateral hind paws. Black arrows indicate the time of spared nerve injury.

To tease apart the role of NFκB in astrocytes vs. peripheral NMSCs following nerve injury, we measured mechanical sensitivity following SNI in IKKdn and littermate controls both on and off Oxy (IKKdn Oxy *n* = 10, IKKdn Off *n* = 10, littermate controls Oxy *n* = 9, littermate controls Off *n* = 8). Male and female mice were tested separately, but the data were pooled because there were no significant sex differences in the previous cohort. We measured mechanical allodynia and paw withdrawal threshold for 2 days prior to SNI and for 3 weeks following peripheral nerve injury. There was no difference in basal mechanical sensitivity in the “off drug” or Oxy groups (Figure 7). Following SNI, all animals developed mechanical allodynia as assessed by paw withdrawal frequency and threshold. In littermate control animals, the mechanical impairment of the ipsilateral paw was maintained through the 3-week testing period. Transgenic IKKdn animals showed alleviation of mechanical sensitivity starting at 1 week following nerve injury (*P* < 0.001). Interestingly, this effect was completely reversed in transgenic animals administered Oxy, which blocks transgene expression peripherally but not centrally. Transgenic animals on Oxy exhibited the same development of mechanical sensitivity as littermate animals following SNI.

In summary, IKKdn suppressed NFκB signaling in both astrocytes and NMSCs in the transgenic mice without Oxy. This resulted in the alleviation of pain behavior following SNI. Oxy inhibited peripheral NMSC expression of IKKdn but left intact central astrocytic expression of IKKdn (Figure 4). As a result, in transgenic mice on Oxy, NFκB signaling is only suppressed centrally in astrocytes. Our data showed that the suppression of NFκB translocation and signaling in nuclei in astrocytes was not sufficient to relieve pain behavior.

## Discussion

The development of neuropathic pain following peripheral nerve damage involves a coordinated peripheral and central response from many cell types, particularly neurons and their associated glia. In the current study, we used transgenic mouse models to dissect the cellular origins of the inflammatory mediators that sensitize neurons following nerve injury. First, the activation of NFκB results in the transcription of a variety of cytokines and chemokines involved in the inflammatory response. Here, we used mice capable suppressing the classical NFκB pathway specifically in GFAP-positive cells. We found that the inhibition of NFκB in GFAP-positive cells temporarily alleviates pain beginning at 1 week and lasts through 3 weeks post-SNI injury in both male and female mice. This is consistent with the study in the chronic constriction injury (CCI) model of nerve injury, in which Fu et al. (2010) also found that transgenic inhibition of NFκB in GFAP-positive cells produced a decrease in pain behavior following nerve injury (Fu et al., 2010).

In addition to the transcription of a host of cytokines and chemokines, the activation of NFκB results in the transcription of COX2, the rate-limiting enzyme converting arachidonic acid to prostaglandins. Following spinal nerve injury, peripheral COX2 is upregulated in a biphasic pattern. There is a brief transient increase in COX2 in Schwann cells from 1 to 3 days post-injury, followed by a persistent increase in macrophages beginning at 7 days and lasting through 4 weeks (Takahashi et al., 2004). Conditional knockout of COX2 in the central and peripheral nervous system using the Nestin promoter is known to be effective in reducing pain behavior in inflammatory pain models (Vardeh et al., 2009); however, its cellular source was previously unknown. In this study, however, we demonstrated that NMSCs are a cellular source of COX2 activation that is necessary for maintaining the mechanical hypersensitivity following nerve-injury.

Prostaglandin E2 (PGE2), as the major downstream product of COX2 activation, is known to play a role in neuropathic pain. Peripheral injection of PGE2 causes pronounced hyperalgesia (Ferreira et al., 1978). By 3 weeks post-nerve injury, the PGE2 receptors EP1 and EP4 are upregulated in macrophages and Schwann cells (Woodhams et al., 2007). Furthermore, endoneurial injection of a COX2 inhibitor following nerve injury has been shown to produce a dose-dependent relief of mechanical hyperalgesia (Syriatowicz et al., 1999). Centrally, there is also a brief transient increase in COX2 mRNA in the deep layers of the dorsal horn starting at 10 h and peaking at 24 h post-SNI (Broom et al., 2004). Enhanced COX2 activation may lead to release of prostaglandins, which can increase neuronal excitability in the spinal cord (Samad et al., 2001). However, the administration of systemic COX2 inhibitors performed better than intrathecal COX2 inhibitors at decreasing pain behavior in neuropathic pain models (Takeda et al., 2005).

Last, we show that transgenic knockout of COX2 specifically in GFAP-positive cells results in the same behavioral phenotype following spared nerve injury as in animals with NFκB suppression in the same cellular population. COX2 conditional knock-out animals showed no significant difference in early pain sensitization at 4 days following spared nerve injury. However, these animals showed temporary inhibition of pain behavior beginning at 1 week in females and 2 weeks in males. This slight delay in males may demonstrate a delay in the activation of inflammatory pathways in GFAP positive cells in males compared to females, however, additional studies with more detailed timing would need to be performed. Mechanical sensitivity developed in the same temporal pattern as in IKKdn transgenic animals, in which NFκB was suppressed. Importantly, neither COX2 knockout nor IKKdn transgenic animals had any changes in basal mechanical nociception compared to wild-type littermates. This supports a great deal of literature showing that glial inflammatory pathways are activated following injury but do not play a role in basal nociception (Myers et al., 2006; Scholz and Woolf, 2007; Campana, 2008; Milligan and Watkins, 2009; Romero-Sandoval et al., 2009; Gosselin et al., 2011).

Glial fibrillary acidic protein-positive cells are in key positions to affect nociceptive neurons. In the peripheral nervous system, small diameter nociceptive fibers are specifically wrapped by NMSCs. Both myelinating and non-myelinating Schwann cells are S100β positive, but only NMSC are GFAP positive (Wewetzer et al., 1997). Schwann cells become activated almost immediately upon nerve injury. Initially, their major role seems to coordinate the recruitment of non-resident macrophages to the injury site (Toews et al., 1998; Siebert et al., 2000). Through their release of cytokines, chemokines, growth factors and matrix metalloproteases, they establish the endoneurial environment. In CNS, following peripheral nerve injury, astrocytes become reactive and produce a host of cytokines and chemokines as well. Furthermore, blockade of astrogliosis by the intrathecal application of fluorocitrate or JNK inhibitors reduces pain behavior (Meller et al., 1994; Milligan et al., 2003; Zhuang et al., 2006).

To tease apart the relative contribution of peripheral and central GFAP-positive glia, we used Oxy, a novel regulator of the tet-Off system, which is functionally similar to doxycycline, except that it does not cross the blood-brain barrier. We have shown that Oxy turns off the tet-Off system of transgene expression peripherally while leaving central transgene expression intact. This finding allowed us to further dissect the cellular origins of our phenotype. In animals which IKKdn expression was suppressed in peripheral NMSCs but not in the CNS, astrocytic expression of IKKdn and therefore suppression of NFκB signaling was not sufficient to alleviate pain behavior flowing SNI. This result suggests that the NFκB signaling in peripheral GFAP-positive glia, the NMSCs are essential in alleviating neuropathic pain in the chronic phase following nerve injuries (Figure 7B).

In both cases, the transgenic suppression of the NFκB or the knockout of COX2 resulted in decreased mechanical sensitivity post-SNI. This finding suggested that suppressing inflammatory pathways in peripheral GFAP^+^ cells alleviates persistent neuropathic pain. In addition to the possible PGE2-related mechanisms discussed above, the suppression of inflammatory signaling in Schwann cells can also lead to a decrease in MCP-1 and a decrease in macrophage recruitment following CCI in the sciatic nerve (Fu et al., 2010; Zhang et al., 2011). Activated NFκB may also regulate the gene expression of extracellular matrix proteins in Schwann cells, thereby influencing their ability to migrate into nerve-injured areas (Anton et al., 1994). Interference with macrophage invasion of the injured area usually reduces the degree of nerve degeneration and pain (Myers et al., 1996). Overall, our findings suggest that peripheral glial signaling play a key role in the maintenance of neuropathic pain. Therapies targeting suppressing GFAP^+^ glia activation using peripheral-acting drugs should be considered following injuries that are known to induce neuropathic pain.

## Data Availability Statement

The original contributions presented in the study are included in the article/supplementary material, further inquiries can be directed to the corresponding author.

## Ethics Statement

The animal study was reviewed and approved by the University of North Carolina at Chapel Hill IACUC.

## Author Contributions

ST and AX conducted the experiments, performed the data analysis, and wrote the manuscript. All authors have read and approved the manuscript, and made contributions to the experimental design and editing of the manuscript.

## Conflict of Interest

The authors declare that the research was conducted in the absence of any commercial or financial relationships that could be construed as a potential conflict of interest.

## Publisher’s Note

All claims expressed in this article are solely those of the authors and do not necessarily represent those of their affiliated organizations, or those of the publisher, the editors and the reviewers. Any product that may be evaluated in this article, or claim that may be made by its manufacturer, is not guaranteed or endorsed by the publisher.
